# The prognostic implication of latitude in uveal melanoma: a nationwide observational cohort study of all patients born in Sweden between 1947 and 1989

**DOI:** 10.1007/s12672-022-00584-0

**Published:** 2022-10-31

**Authors:** Gustav Stålhammar, Pete A. Williams, Tomas Landelius

**Affiliations:** 1 Department of Clinical Neuroscience, Division of Eye and Vision, St. Erik Eye Hospital, Karolinska Institutet, Eugeniavägen 12, 171 64 Stockholm, Sweden; 2 Swedish Meteorological and Hydrological Institute (SMHI), Norrköping, Sweden

## Abstract

**Background:**

The incidence of uveal melanoma increases with latitude. In this study, we examine the importance of latitude for uveal melanoma prognosis.

**Methods:**

All uveal melanoma patients born in Sweden between 1947 and 1990 were included (*n* = 745). The latitude of patients’ birthplaces and home counties at the time of uveal melanoma diagnosis were collected. For all latitudes, data on sunlight and UV intensity parameters, temperature, daytime length variations, and socioeconomic factors were added. The prognostic implication of birthplace latitude and of moving > 1 degree of latitude was examined with multivariate Cox regressions and competing risk analyses.

**Findings:**

There were no significant differences in patient sex, age, tumor size, T-category, or BAP-1 immunoexpression between patients born in the south, central or northern regions of Sweden. Decreasing birthplace latitude was a predictor of uveal melanoma-related mortality in multivariate Cox regression. Patients that were born in southern regions or moved > 1 degree south between birth and diagnosis had higher incidence of uveal melanoma-related mortality in competing risk analysis. The sum of yearly sunshine hours, global sunlight radiation, average daily ultraviolet light intensity, average annual temperature, or net wealth were not predictors of uveal melanoma-related mortality.

**Interpretation:**

Latitude is a prognostic factor in uveal melanoma. This does not seem to be related to variations in patient or tumor characteristics at presentation, in management, in sunlight intensity, in ultraviolet light irradiance, in temperature, or in wealth. Future studies should examine if periodical changes in daylight hours or other factors could explain the prognostic implication.

**Supplementary Information:**

The online version contains supplementary material available at 10.1007/s12672-022-00584-0.

## Introduction

The incidence of uveal melanoma (UM) increases with increasing latitude [1, 2]. Ongoing research has attributed this to differences in the eye color of populations living at southern and northern latitudes [1]. Blue or gray iris color has been associated with increased prevalence of choroidal melanocytic lesions including uveal melanoma, in addition to an increased risk for metastatic death [3]. However, the incidence of UM is greater at northern latitudes even when only non-Hispanic whites are included [2]. Other groups have pointed to the partially protective effect of solar irradiance on tissues not directly exposed to sunlight by increased synthesis of vitamin D and other antioxidants, suggesting that the greater incidence on high latitudes rather depends on a relative absence of sunlight [2, 4]. It is likely that this may be an over-simplification: The yearly total direct normal irradiance (DNI), which is equal to the extraterrestrial solar irradiance above the atmosphere minus atmospheric losses is influenced by several other local factors than latitude, including altitude, local climate, cloud cover, moisture, and atmospheric particles [5]. Therefore, the DNI is higher in southern Spain than at the equator; and higher in Colorado than in Florida [6]. The age-standardized UM incidence rates in Australia, marked by a predominantly Caucasian population, is similar to the incidence in the north of Europe –where the DNI is much lower [7]. Consequently, neither differences in eye color nor the protective effect of sunlight are fully satisfying explanations for the greater incidence of UM on higher latitudes.

Sweden is situated in the north of Europe and stretches between 55 and 70 degrees of latitude. Available data indicate that the population is genetically homogenous with little to no variation in haplotype and Y-chromosome short tandem repeats between different regions [8, 9]. As in other parts of Scandinavia, 60 to 70% of individuals residing in Sweden have blue or intermediate eyes, whereas a minority have brown eyes [10]. If only persons that are born in Sweden are included, the proportion of individuals with blue eyes will be even higher. Each Swedish citizen is identifiable with a unique personal identity number (PIN). This number includes information about the day of birth and sex. For persons born between January 1st, 1947, and December 31st, 1989, the PIN also contained information about birthplace county or if the person was born outside Sweden. This allows for a selection of native patients with known birthplace latitude. The initiation, growth and progression of UM is a slow process, with metastatic seeding and prognostically relevant mutations estimated to occur one to four decades before a medium-sized tumor is diagnosed [11]. This might make the latitude on which a patient is born and raised relevant for UM prognosis, as it is for incidence.

Swedish crude and age-standardized UM incidence rates, which are among the world’s highest, have remained stable during the last 50 years at 6.5–11.6 and 5.6–9.6 cases/million/year, respectively [12]. The management of Swedish UM patients has been highly centralized since the 1960s, with St. Erik Eye Hospital in Stockholm being responsible for the diagnosis of all patients. Consequently, the hospital’s patient registry has been estimated to capture more than 95% of all UMs in the country [13]. Likewise, the management of diabetic retinopathy (DRP) and age-related macular degeneration (AMD), during which many asymptomatic tumors are discovered, has been similar throughout the country as a result of a single-payer healthcare system and laws dictating equal and universal access to healthcare. When registries and guidelines for the diagnosis, screening, intervention, and follow-up of cataract, DRP and AMD have been devised, they have been introduced simultaneously on a national level [14–16].

Together with the Swedish Radiation Safety Authority and the Swedish Environmental Protection Agency, the Swedish Meteorological and Hydrological Institute (SMHI) have set up the STRANG mesoscale model that estimates global radiation, photosynthetically active radiation, UV radiation, and DNI at a horizontal resolution of 2.5 to 22 km^2^ and a temporal resolution of one hour. The model has been validated with data from a network of SMHIs solar sensors.

Considering the observed differences in incidence, we hypothesize that there is also a prognostic implication of latitude [1, 2]. The combination of a large country that spans 15 degrees of latitude; a homogenous population; a centralized diagnostic procedure and follow-up; traceability of birthplace county; and access to solar intensity data with high geospatial resolution, allows for a unique setting to test this hypothesis.

### Methods

All Swedish UM patients born between January 1st, 1947, and December 31st, 1989, were included in the study (*n* = 745). Patients born outside Sweden, as identified by their PINs, were not included. The latitude and longitude (according to the World Geodetic System 1984, WGS84) of the largest population centers in each included patient’s birthplace county was collected from The Swedish Mapping, Cadastral and Land Registration Authority (SMCLRA). From patient journals, referral letters and notes in our patient registry, we also collected data on the patients’ home counties at the time of UM diagnosis. The latitude of the largest population centers in each home county was then obtained from SMCLRA. Only county-level geographic information was collected, and no home addresses, zip-codes, phone numbers, names or other contact details to individual patients.

From STRANG, we collected the following solar irradiance parameters from the 2.5 to 22 km^2^ patch that included each patients birthplace: (1) the yearly global horizontal irradiance (GHI) which is the sum of DNI and the diffuse horizontal irradiance by light scattering in the atmosphere and is measured in kWh/m^2^; and (2) the average daily International Commission on Illumination (CIE)-weighted ultraviolet light (UV) irradiance as measured in mWh/m^2^. From SMHI, we also obtained data on (3) the duration of a year’s longest and shortest day, (4) the number of yearly sunlight hours with an intensity of > 120 W/m^2^, which is the definition of sunshine according to the World Meteorological Organization[17], and (5) the average annual temperature on each patient’s birthplace. Year 2000 and 2010 were used as index years for data from STRANG, whereas average values over the entire period 1961 to 1990 were used for all the remaining parameters. Data on the net wealth per inhabitant across Swedish municipalities and Gross Regional Products (GRP) were collected from Statistics Sweden [18].

Clinicopathological- and survival data including patient age, sex, tumor diameter and tumor thickness, time of death, and cause of death was collected from our patient registry. All patients were diagnosed at St. Erik Eye Hospital, were treated with plaque brachytherapy or enucleation within 6 weeks from diagnosis, and were followed-up according to the same routine regardless of place of residence. To reduce the number of classification errors, e.g., death from metastatic UM coded as death from metastatic cutaneous melanoma, the registry had been crosschecked against other diagnoses in the national Cancer Registry, against results from metastatic screening with ultrasound or computed tomography (CT) of the abdomen/liver every 6 months for a minimum of 5 years after diagnosis, and against hospital medical records, as described previously [19–21]. The study follows the tenets of the Declaration of Helsinki and the research group's internal data security policy for personal data. Ethical permission was obtained from the Swedish Ethical Review Authority (record number 2022-00930-02).

### BAP-1 immunohistochemistry

The level of BAP-1 expression was analyzed in tumors from all patients with available formalin-fixed and paraffin-embedded (FFPE) enucleated eyes in the archives of the St. Erik Ophthalmic Pathology Laboratory. Each tumor was cut into a 4 μm section, pretreated in ethylenediaminetetraacetic acid (EDTA) buffer at pH 9.0 for 20 min, and incubated with mouse monoclonal antibodies against BAP-1 at dilution 1:40 (clone C-4; Santa Cruz Biotechnology, Dallas, TX) and a red chromogen secondary antibody kit (Leica Biosystems, Nußloch, Baden-Wurttemberg, Germany), and finally counterstained with hematoxylin and rinsed with deionized water. The deparaffinization, pretreatment, primary staining, secondary staining, and counter-staining steps were run in a Bond III automated IHC/ ISH stainer (Leica, Wetzlar, Germany). The dilutions had been gradually titrated until optimal staining was achieved, according to manual control. The level of nuclear BAP-1 expression was assessed by GS according to a previously described method [22]. For a tumor to be classified as BAP-1 positive, at least 33% of tumor cell nuclei had to be positively stained, and accumulation of chromogen in nucleoli or similar did not suffice [23].

### Statistical methods

**Fig. 1:**
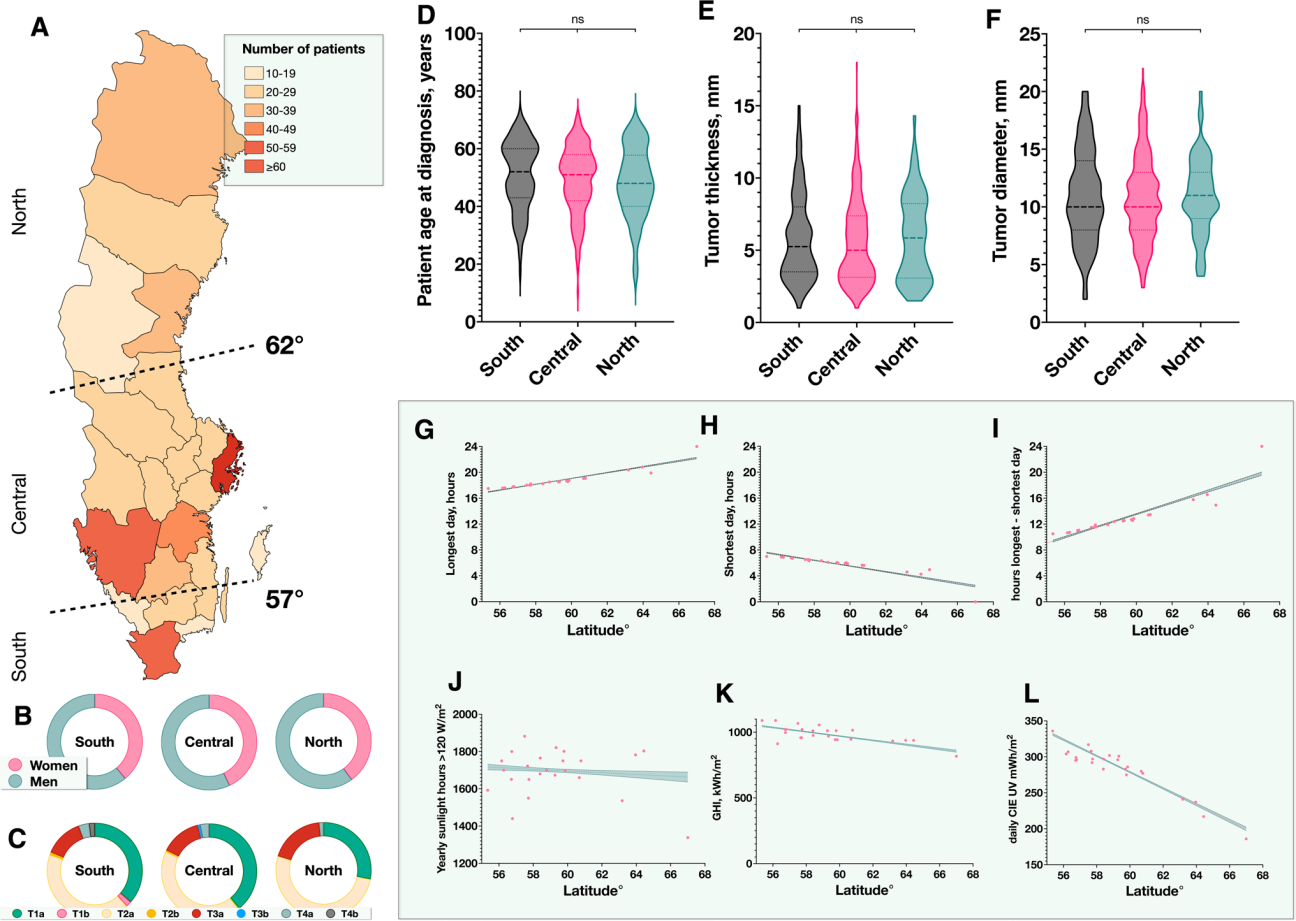
Characteristics of patients, tumors and sunlight intensity across regions and latitudes. **A** Number of patients born in the southern, central, and northern regions and individual counties. **B** The distribution of patient sex was similar in all three regions (χ^2^ *P* = 0.66). **C** Similarly, there were no significant differences in distribution of AJCC T-categories across the regions (χ^2^ *P* = 0.48). **D** to **F** There were no significant differences in patient age at diagnosis (Kruskal–Wallis *P* = 0.21), tumor thickness (*P* = 0.39) or tumor diameter (*P* = 0.86) across the three regions. **G** The duration of the longest day in a year increased with a slope coefficient of 0.45 h per degree of latitude (R^2^ = 0.86, *P* < 0.001). **H** The duration of the shortest day in a year decreased with a slope coefficient of − 0.44 h per degree of latitude (R^2^ = 0.78, *P* < 0.001). **I** Consequently, the difference between the longest and shortest day in a year increased with latitude (R^2^ = 0.82, *P* < 0.001). **J** The annual hours with an intensity of > 120 W/m^2^ decreased with 5 h/year per degree of latitude (R^2^ = 0.01, *P* = 0.005). **k** Similarly, the GHI decreased with 16 kWh/m^2^ per increasing degree of latitude (R^2^ = 0.58, *P* < 0.001). **L** The average daily CIE UV decreased with -11 mWh/m^2^ per increasing degree of latitude (R^2^ = 0.87, *P* < 0.001). *AJCC* American Joint Committee on Cancer, *ns* non-significant, *GHI* annual global horizontal irradiance, *UV* ultraviolet light, *CIE UV* CIE (International Commission on Illumination)-weighted UV irradiance

*P* values below 0.05 were considered statistically significant, all *P* values being two-sided. For tests of continuous variables that did not deviate significantly from normal distribution (Shapiro–Wilk test *P* > 0.05) Student’s *t*-tests were used. For non-parametrical data, Mann–Whitney *U* tests were used. For comparisons of continuous variables across three categories or more, we used the Kruskal–Wallis test. In comparisons of categorical variables, we used contingency tables and Pearson chi-square (χ^2^) tests (if all fields had a sample of > 5) or Fisher’s exact tests (if any field had a sample of < 5). Linear regressions were used to evaluate solar irradiance parameters and temperature in relation to latitude. Uni- and multivariate Cox regressions were used to evaluate the association between patient, tumor- and, latitude-, longitude-, temperature-, solar irradiance-, and net wealth per capita parameters, and the hazard ratio (HR) for UM-related mortality. Variates that were significant in univariate regression were entered into multivariate regression, except factors that were inseparable from each other, e.g., tumor dimensions and American Joint Committee on Cancer (AJCC) primary tumor T-category 1 to 4; and latitude and the variance in the length of day and night over a year.[24, 25] Latitude and covariates relating to solar irradiance could be entered simultaneously into multivariate regressions if significant in univariate models, since they are not necessarily inseparable from each other. For patients born in the south, central and north region of Sweden and for patients moving south or north between birth and UM diagnosis, we plotted cumulative incidence function estimates of UM-related mortality from competing risks data with the cmprsk package for R, and the equality of survival distributions was tested with Gray’s test for equality [26]. The southernmost region was defined as a latitude of < 57°, which includes the southernmost and relatively populous county of Skåne including Malmö. The central region was defined as a latitude of ≥ 57° to < 62° which includes the two largest cities of the country (Stockholm and Göteborg). The northernmost region was defined as a latitude of ≥ 62°. The map in Fig. 1 was made with mapchart.net under a Creative Commons Attribution-ShareAlike 4.0 International License. All statistical analyses were performed using IBM SPSS statistics version 27 (Armonk, NY, USA) and GraphPad Prism version 9.3.0 (San Diego, CA, USA).

## Results

### Descriptive statistics

Of the 745 included patients, 311 (42%) were women and 434 (58%) were men. Their median age at diagnosis was 51 years (interquartile range, IQR, 16). Tumors had a mean apical thickness of 5.7 mm (standard deviation, SD, 2.9) and a mean diameter of 10.9 mm (SD 3.7). 155 patients (21%) had been born in the southern region, 495 patients (66%) in the central region, and the remaining 96 patients (13%) in the northern region (Fig. 1A). Two hundred and thirteen patients had deceased at the time of data collection, of which 163 (77%) had deceased from metastatic uveal melanoma. The median follow-up for the 564 survivors was 7.3 years (IQR 9.1) and for all patients including deceased 5.9 years (IQR 7.9, Table 1).

**Table 1:** Demographics and clinical features of study patients

n	745
Age at diagnosis, median (IQR)	51 (16)
Sex, n (%)	
Female	311 (42)
Male	434 (58)
Tumor thickness at diagnosis, mean mm (SD)	5.7 (2.9)
Tumor diameter at diagnosis, mean mm (SD)	10.9 (3.7)
Birthplace latitude, mean ° (SD)	59.1 (2.7)
Patient birthplace region, n (%)	
South	155 (21)
Central	495 (66)
North	96 (13)
Birthplace longitude, mean ° (SD)	15.7 (2.3)
Birthplace county, n (%)	
Blekinge	12 (1.6)
Gotland	11 (1.5)
Gävleborg	26 (3.5)
Göteborg och Bohus	51 (6.8)
Halland	19 (2.6)
Jämtland	12 (1.6)
Jönköping	39 (5.2)
Kalmar	22 (3)
Kopparberg	25 (3.4)
Kristianstad	20 (2.7)
Kronoberg	22 (3)
Malmöhus	60 (8.1)
Norrbotten	30 (4)
Skaraborg	15 (2)
Stockholm	133 (17.9)
Södermanland	25 (3.4)
Uppsala	24 (3.2)
Värmland	22 (3)
Västerbotten	23 (3.1)
Västernorrland	31 (4.2)
Västmanland	24 (3.2)
Älvsborg	35 (4.7)
Örebro	21 (2.8)
Östergötland	43 (5.8)
Median follow-up for survivors, years (IQR)	7.3 (9.1)

*IQR* Interquartile range, *SD* standard deviation

### Patient, tumor, and sunlight characteristics versus latitude

The distribution of patient sex and AJCC primary tumor T-category were independent of region (χ^2^ *P* = 0.66 and *P* = 0.48, respectively. Figures 1B and C). Similarly, there were no significant differences in patient age, tumor thickness or tumor diameter at diagnosis between patients born in the three regions (Kruskal–Wallis *P* ≥ 0.21, Fig. 1D–F).

Immunohistochemical expression levels of BAP-1 were available for 134 patients treated with primary enucleation. There were no differences in the proportion of BAP-1 negative tumors across the three regions (χ^2^ *P* = 0.91). In binary logistic regression, BAP-1 negativity was not associated with latitude degree (odds ratio 0.99, 95% CI 0.85 to 1.16, *P* = 0.93).

In linear regressions, the duration of the longest day and night in a year increased with the latitude (*P* < 0.001, Figs. 1G and H). The difference between the longest and shortest day in a year increased with the latitude from 9 h in the southernmost region to 24 h in the northernmost (R^2^ = 0.82, *P* < 0.001, Fig. 1I). The sum of yearly sunlight hours with an intensity of > 120 W/m^2^ decreased with 5 h/year per degree of latitude (R^2^ = 0.01, *P* = 0.005, Fig. 1J). Similarly, the yearly global sunlight radiation decreased with 16 kWh/m^2^ per increasing degree of latitude (R^2^ = 0.58, *P* < 0.001, Fig. 1K). The average daily CIE UV decreased with -11 mWh/m^2^ per increasing degree of latitude (R^2^ = 0.87, *P* < 0.001, Fig. 1L). Lastly, the average annual temperature ranged from 8.5 °C in the south to −2 °C in the north and decreased with − 0.7 °C per degree of latitude (R^2^ = 0.80, *P* < 0.001).

The crude UM incidence for the period 1974 to 1989 had no linear relation with birthplace latitude, CIE UV, or with diffuse CIE UV (*P* > 0.22). The net wealth had a negative correlation with increasing degree of latitude (R^2^ = 0.24, *P* = 0.02), and was lowest in the northern region (Kruskal–Wallis *P* = 0.007). However, the GRP per capita was unevenly distributed over the country and followed no specific pattern in relation to latitude (supplementary Fig. 1).

### Survival

In univariate Cox regressions, increasing patient age at diagnosis, tumor diameter, tumor thickness, AJCC T-category 1 to 4, and duration of a year’s shortest day were associated with increased risk for UM-related mortality, whereas increasing birthplace latitude (per degree), birthplace region (southern, central, or northern), increasing duration of a year’s longest day, and increasing difference between a year’s longest and shortest day were associated with reduced risk. The number of yearly sunlight hours, the yearly global sun radiation, the average daily UV intensity, average annual temperature, and net wealth per capita were not associated with UM-related mortality.

**Fig. 2:**
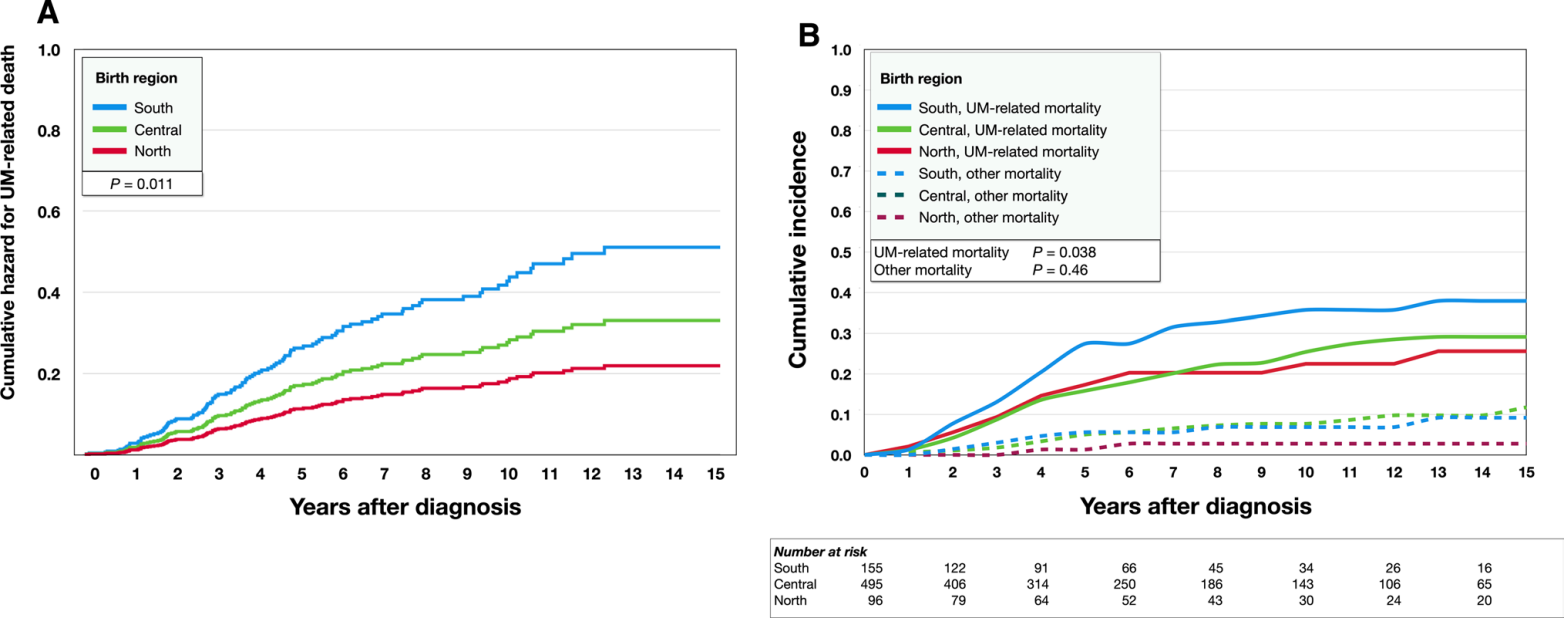
Survival curves. **A** Cumulative Cox regression hazard for uveal melanoma (UM)-related death across the southern, central, and northern regions. Adjusted for patient age at diagnosis, tumor diameter and tumor thickness. **B** Cumulative incidence of UM-related mortality and mortality from other causes. UM, uveal melanoma

In multivariate analysis with patient age, tumor diameter, thickness, and birthplace region as covariates, all except patient age retained their significance (Table 2, Fig. 2A).

**Table 2:** Cox regressions, hazard for uveal melanoma-related death

	B	S.E	Wald	*P*	Exp(B)	95% CI lower	95% CI upper
Univariate							
Patient sex, male vs female	0.04	0.16	0.07	0.797	1.04	0.76	1.42
Patient age at diagnosis^a^	0.14	0.07	4.22	0.040	1.15	1.01	1.32
Tumor diameter^b^	0.14	0.03	33.88	< 0.0001	1.15	1.10	1.21
Tumor thickness^b^	0.18	0.03	38.96	< 0.0001	1.19	1.13	1.26
AJCC T-category 1 to 4	0.58	0.10	34.17	< 0.0001	1.79	1.47	2.17
Birthplace region^c^	− 0.34	0.14	6.00	0.014	0.71	0.55	0.94
Birthplace latitude^d^	− 0.07	0.03	5.42	0.020	0.93	0.87	0.99
Longest day^e^	− 0.18	0.08	5.40	0.020	0.84	0.72	0.97
Shortest day^e^	0.18	0.08	5.29	0.021	1.19	1.03	1.40
Difference longest to shortest day^e^	− 0.09	0.04	5.37	0.021	0.91	0.85	0.99
Yearly sunlight hours^f^	0.00	0.00	0.16	0.690	1.00	1.00	1.00
Yearly sunlight hours, rounded^g^	0.02	0.06	0.14	0.705	1.02	0.91	1.15
GHI^h^	0.00	0.00	2.11	0.146	1.00	1.00	1.01
Average CIE UV^i^	0.00	0.00	2.44	0.118	1.00	1.00	1.01
Average annual temperature^j^	0.08	0.04	3.48	0.062	1.08	1.00	1.18
Net wealth per capita	0.00	0.00	0.17	0.676	1.00	1.00	1.00
Multivariate							
Patient age at diagnosis^a^	0.12	0.10	1.50	0.221	1.12	0.93	1.36
Tumor diameter^b^	0.10	0.03	11.32	0.001	1.10	1.04	1.17
Tumor thickness^b^	0.11	0.04	8.95	0.003	1.12	1.04	1.20
Birthplace latitude^c^	− 0.43	0.17	6.44	0.011	0.65	0.47	0.91

*AJCC* American Joint Committee on Cancer

^a^Per increasing decade. ^b^Per increasing mm. ^c^Per increasing step from 55–58°, 59–62° through ≥ 63°. ^d^Per increasing degree of latitude. ^e^Per increased hour. ^f^Per increased hour with a light intensity > 120 W/m^2^. ^g^Divided by 100 and rounded to nearest integer. ^h^Per increased kWh/m^2^ of yearly global horizontal irradiance (GHI). ^i^Per increased mWh/m^2^ of average daily ultraviolet light (UV) irradiance weighted according to recommendations from the International Commission on Illumination (CIE). ^j^Per increasing degree Celsius

The cumulative incidence of UM-related mortality was highest for patients born in the southern region, lower for patients born in the central region and even lower for patients born in the northern region (Gray’s test for equality *P* = 0.038, Fig. 2B). The 5, 10, and 15-year incidences of UM-related mortality was 27, 36 and 38% for patients born in the southern region, 16, 25 and 29% for patients born in the central region, and 17, 22 and 26% for patients born in the northern region (Table 3). There was no significant difference in the cumulative incidence of mortality from other causes (*P* = 0.46).

**Table 3:** Cumulative incidence of uveal melanoma-related mortality

Year after diagnosis	Region		
	South (%)	Central (%)	North (%)
5	27	16	17
10	36	25	22
15	38	29	26

### Prognostic implication of having moved to another latitude

Next, we aimed to examine the prognostic implication of moving across latitudes. This could help answer if the better prognosis on northern latitudes was related to patient characteristics or to an environmental factor: If an environmental factor influenced the incidence of UM-related mortality, we hypothesized that patients moving south between birth and UM diagnosis would have a worse prognosis than patients moving north. For 586 of the patients, the home county at the time of UM diagnosis could be determined. Patients that had moved south with at least one degree of latitude (*n* = 97) were compared with patients that had moved north with at least one degree of latitude (*n* = 76), the remaining 413 patients resided within ± 1 degree of latitude from their birthplace at the time of UM diagnosis.

**Fig. 3:**
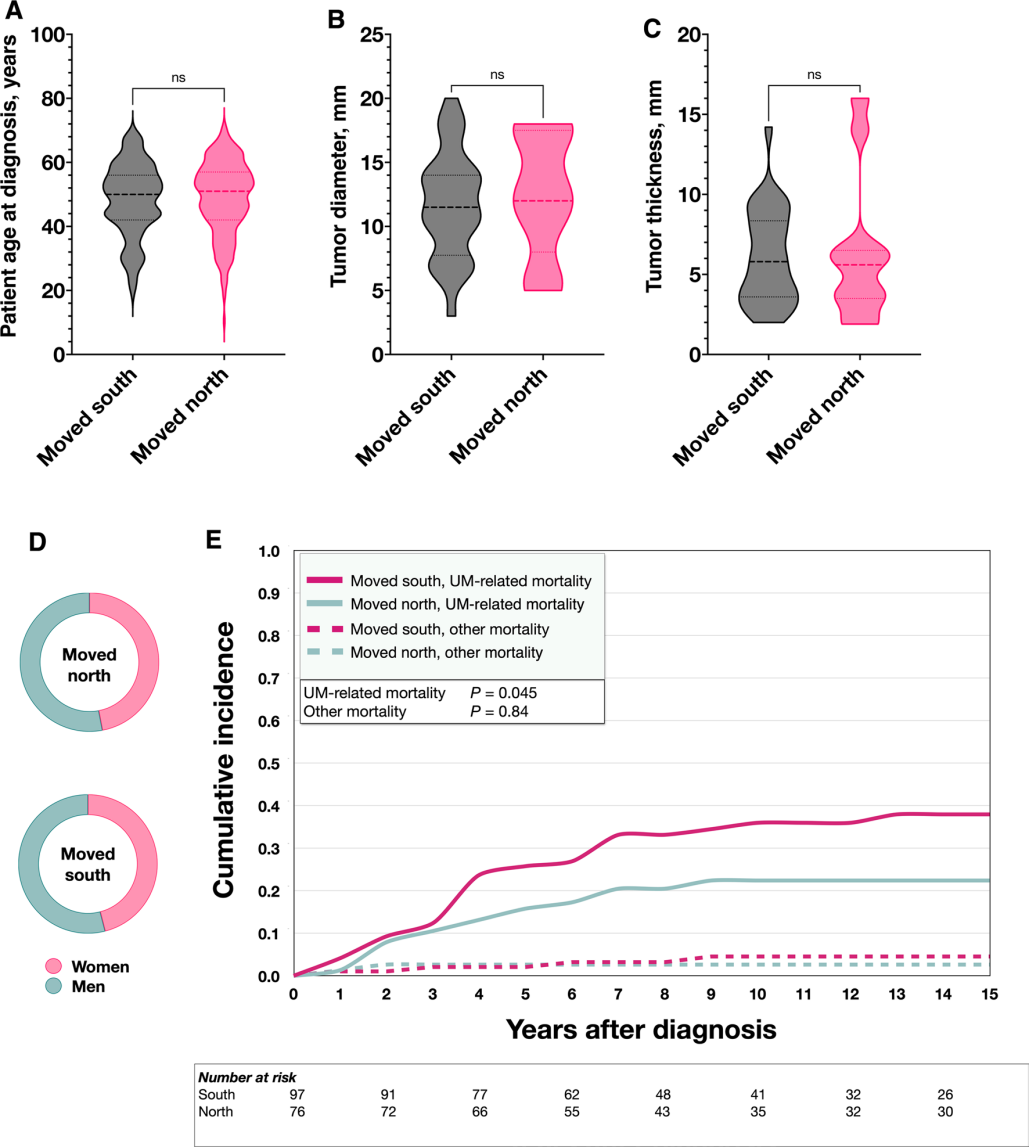
Characteristics and survival of patients moving south versus north. At UM diagnosis, 97 and 76 patients resided > 1 degree of latitude south and north of their birthplace, respectively. **A** to **D** There were no significant differences in patient age at diagnosis (Mann–Whitney *U P* = 0.44), tumor diameter (*P* = 0.72), tumor thickness (*P* = 0.66) or sex (χ^2^ *P* = 0.90) between patients that had moved south or north. **E** In cumulative incidence analysis, patients that had moved south had significantly higher incidence of UM-related mortality than patients that had moved north. UM, uveal melanoma

There were no significant differences in patient age at diagnosis, tumor diameter, tumor thickness (Mann–Whitney *U P* > 0.44, Fig. 3A –C) or patient sex (χ^2^ *P* = 0.90, Fig. 3D) between patients that had moved > 1 degree of latitude south or north. In cumulative incidence analysis, patients that had moved south had significantly higher incidence of UM-related mortality than patients that had moved north (Gray’s test for equality *P* = 0.045, Fig. 3E).

In conclusion, latitude appears to be a prognostic factor in UM, independent of patient age, sex, tumor diameter, tumor thickness, AJCC T-category, BAP-1 expression, to the quantity of sunlight or UV, or to temperature. This is further corroborated by the demonstration of a better prognosis for patients that moved north between birth and UM diagnosis.

## Discussion

In this study, we demonstrate that increasing birthplace latitude is associated with a lower hazard for UM-related mortality, independent of patient age, tumor diameter, thickness and AJCC T-category. Further, patients that had moved at least one degree of latitude north from their birthplace latitude at the time of UM diagnosis had significantly lower incidence of UM-related mortality than patients that had moved south. Given our data, latitude appears to be a prognostic factor for UM in Sweden.

The reason for the association between latitude and prognosis does not seem to be directly related to the quantity of sunlight or UV, or to temperature. And as also demonstrated in this study, the prognostic implication cannot be explained by differences in patient age, tumor size, ciliary body involvement, extraocular extension, BAP-1 expression, in diagnostic procedures or in management of the included patients. It is therefore reasonable to hypothesize that another unknown causal factor is in play. The variations in the number of daylight hours over a year were indeed significantly associated with UM-related mortality, but they cannot be separated from the effect of latitude as they are its direct consequence.

One may be tempted to propose that this factor, regardless of its nature, leads to a higher prevalence of indeterminate pigmented choroidal tumors in patients born on northern latitudes, and that some of these are diagnosed and treated as UM even though they would not have progressed to melanomas if left untreated; or that differences in diabetic screening practices, cataract surgery numbers, awareness among ophthalmologists or similar patterns could lead to earlier detection of small tumors and thereby a better prognosis. This would explain both the higher incidence and better survival in the north. Arguing against this, our data shows that tumors in the north were not smaller at diagnosis: Higher latitude is a predictor of lower hazard for UM-related mortality regardless of tumor size and patient age. Future studies should therefore examine if intrinsic tumor factors could be the drivers behind the survival differences. *e.g*., if cytogenetic, genetic or epigenetic changes associated with a more favorable prognosis such as disomy 3, or mutations in *EIF1AX* or *SF3B1* are relatively more common in the north -even though tumor dimensions are similar [22, 27–30]. Alternatively, environmental factors other than sunshine UV, and temperature might have an influence over UM prognosis. The northernmost region is markedly more rural, which may be associated with differences in lifestyle, exercise and food intake that are not necessarily connected to net wealth. Diamantopoulou et al. recently reported that the number of circulating breast cancer tumor cells in the bloodstream increases at night and decreases in daytime, and that circulating tumor cells generated during daytime are devoid of metastatic ability [31]. This suggests that the circadian rhythm can play an important role in cancer progression. Great variations in daytime length over the year, a hallmark of the high north, may disrupt the circadian rhythm which in turn affects multiple other systemic and molecular mechanisms, including the melatonin, leptin, and glucocorticoid axes, the gut microbiome, and energy metabolism [32]. Importantly, deregulation of the circadian rhythm increases the risk of obesity, which in turn may have direct consequences for UM survival [32–34]. This and other aspects of the circadian rhythm could at least partially be driving the survival differences found herein [35–37]. However, a dedicated experiment would have to be conducted, e.g., to examine the prognosis of UM patients living on the same latitude with and without circadian disruption, to separate this effect from other consequences of latitude. Oral supplement with high doses of melatonin has been proposed as an adjuvant treatment for uveal melanoma, with a Swedish clinical trial currently being launched (clinicaltrial.gov identifier NCT05502900) [38].

Increased prevalence of blue eyes or decreased solar irradiation are *not* satisfactory explanations for neither the higher incidence of UM nor a better survival on northern latitudes. To date, there is no data supporting a higher prevalence of blue eyes among Swedish citizens born on higher latitudes. Further, according to official statistics, the proportion of the population born outside Sweden has ranged from 2 to 5% during the studied period, and was even lower than that in the preceding decades [39]. Additionally, a vast majority of immigrants came from the surrounding Scandinavian countries and northern Europe [40]. The Nordic countries are similar in terms of ethnicity, economy, welfare and health care as well as in life expectancy and distribution of overall disease burden [41, 42]. Children of immigrants would be eligible for inclusion, but their effect on the distribution of blue eyes would therefore be marginal even if they were overrepresented in one of the examined regions. Future studies might reveal that the prevalence of blue eyes is indeed higher in the north than in the south of Sweden, and that it is a probable cause for a higher incidence. Even so, it would not explain why UM patients born on these latitudes have a better prognosis. Similarly, if there truly was a protective effect from increased quantities of solar irradiation, one could have expect a better survival on southern latitudes in this material, which would be in line with the beneficial survival effect of Vitamin D levels in several other cancers [43, 44]. However, this scenario is literally the opposite of what was shown here and the theory of a UM-protective effect from indirect solar exposure could perhaps be considered falsified.

This study has several limitations. Foremost, we could exclude several variables as the cause for the prognostic implication of latitude, but based on the available data, none could be verified as the true causal factor. We propose that future studies seek to clarify if circadian rhythm disruption, or any other factor, can explain why patients on northern latitudes have better prognosis. If such a factor can be identified, it could have important consequences on both management and survival of our patients. Secondly, this data was retrospective and non-randomized, which limits control over confounding factors. Thirdly, only 134 of 745 patients had tissue for BAP-1 immunohistochemistry available, limiting the generalizability of this analysis. Fourthly, even though Sweden stretches out over a span of latitudes, it is entirely located in the northern end of Europe, and we do not know that the results apply to more southern latitudes.

In conclusion, we demonstrate that latitude is a prognostic factor in UM. This association does not seem to be related to differences in patient age, sex, tumor diameter, tumor thickness, BAP-1 expression, AJCC T-category, to the quantity of sunlight or UV, to temperature, or to differences in wealth. Immigrants were excluded and all patients were diagnosed at the same institution, making it unlikely that major differences in patient characteristics and clinical management across latitudes confound the results. Future studies should examine if variations in daylight hours, circadian disruption or other environmental factors can explain the prognostic implication.

## Supplementary Information

Supplementary file 1 (XLSX 69 KB)

Supplementary file 2 (PDF 3991 KB)

## Data Availability

The raw data for this study is provided as a supplementary file.

## Abbreviations

AJCC: American Joint Committee on Cancer
AMD: Age-related macular degeneration
CIE UV: International Commission on Illumination-weighted ultraviolet light irradiance
DNI: Yearly total direct normal irradiance
DRP: Diabetic retinopathy
IQR: Interquartile range
PIN: Personal identity number
SD: Standard deviation
SMHI: Swedish Meteorological and Hydrological Institute
UM: Uveal melanoma
UV: Ultraviolet light
WGS84: World Geodetic System 1984
