# Supplementary material for: The prognostic implication of latitude in uveal melanoma: a nationwide observational cohort study of all patients born in Sweden between 1947 and 1989
Source: Discov Oncol. 2022 Oct 31;13:116. doi: 10.1007/s12672-022-00584-0 (PMC9618472; doi:10.1007/s12672-022-00584-0)
Supplement: Supplementary file 2 — (PDF 3991 KB) [file 12672_2022_584_MOESM2_ESM.pdf]

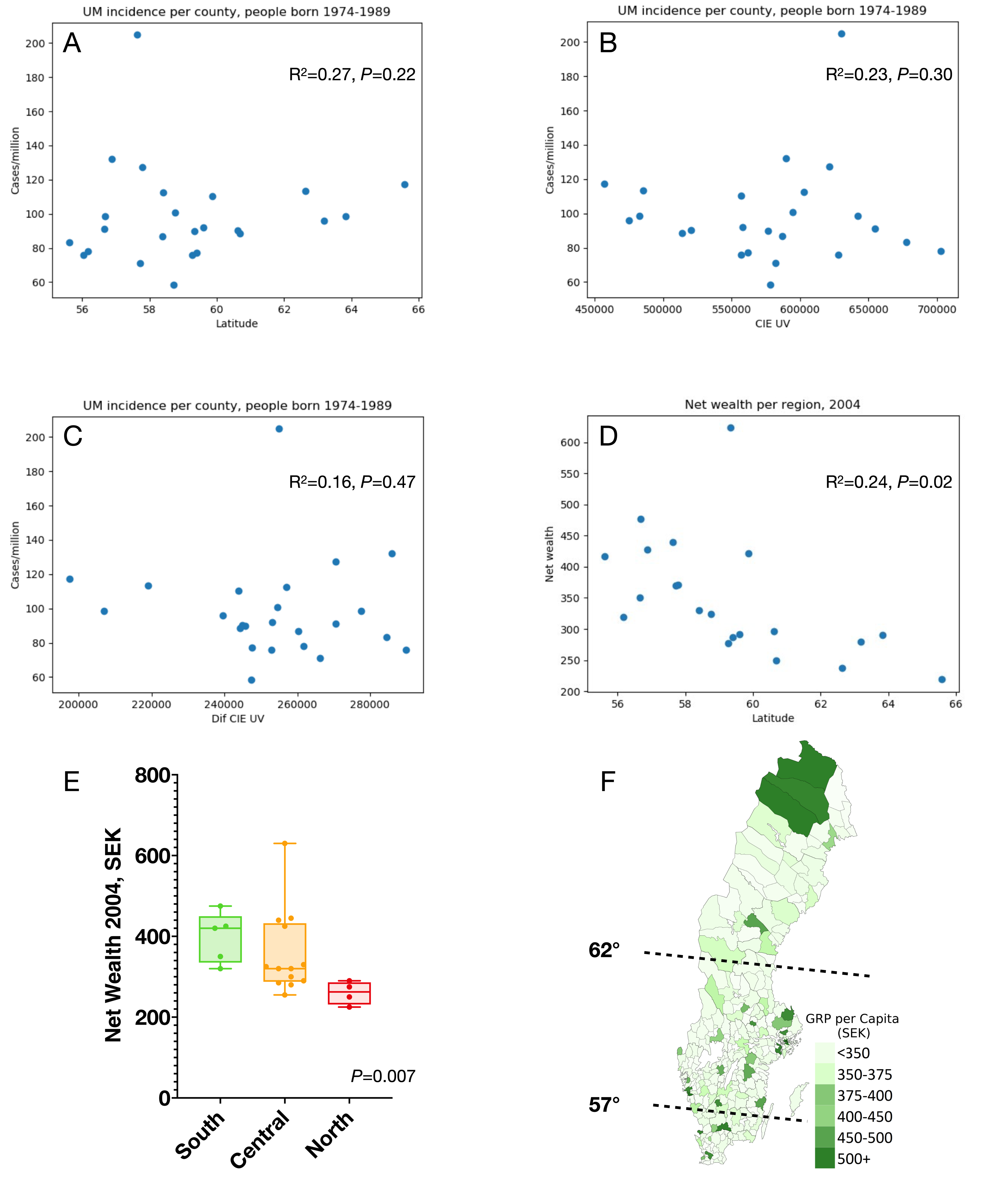


**Supplementary figure 1.** Uveal melanoma incidence for patients born 1974 to 1989 and wealth in relation to latitude and UV irradiation. A) Uveal melanoma incidence vs. latitude. B) Uveal melanoma incidence vs. CIE UV. C) Uveal melanoma incidence rate vs. Dif CIE UV. D) Net wealth (in 1000 SEK per inhabitant) vs. latitude. E) Net wealth per region. F) GRP per municipality.

UV, Ultraviolet. CIE UV, International Commission on Illumination-weighted ultraviolet light irradiance. Dif CIE UV, Diffuse CIE UV. GRP, Gross Regional Product.
